# Exercises in activating lymphatic system on fluid overload symptoms, abnormal weight gains, and physical functions among patients with heart failure: A randomized controlled trial

**DOI:** 10.3389/fcvm.2023.1094805

**Published:** 2023-04-11

**Authors:** Yuan Li, Qingtong Meng, Biru Luo, Minlu Li, Jinbo Fang, Sarah R. Allred, Mei Rosemary Fu

**Affiliations:** ^1^ Department of Neonatology, West China Second University Hospital, Sichuan University, Chengdu, China; ^2^ Department of Nursing, West China Second University Hospital, Sichuan University, Chengdu, China; ^3^ West China School of Nursing, Sichuan University, Chengdu, China; ^4^ Key Laboratory of Birth Defects and Related Diseases of Women and Children (Sichuan University), Ministry of Education, Chengdu, China; ^5^ Department of Cardiology, Shenzhen People's Hospital, Shenzhen, China; ^6^ General Ward of Neurology, West China Hospital, Sichuan University, Chengdu, China; ^7^ Department of Psychology and Health Sciences, The State University of New Jersey, Camden, NJ, United States; ^8^ School of Nursing, George Washington University, Washington, DC, United States

**Keywords:** heart failure, lymphatic system, fluid overload, exercise, symptoms

## Abstract

**Background:**

Fluid overload remains a vexing problem in management of heart failure. The lymphatic system that plays the central role in fluid homeostasis has recently been explored as a potential target to counteract tissue fluid overload. The goal of the study was to evaluate the preliminary effects of exercises in activating lymphatic system on fluid overload symptoms, abnormal weight gains, and physical functions for patients with heart failure.

**Methods and results:**

A pilot, pre- and post-test, randomized clinical trial was conducted to recruit a total of 66 patients who were randomized to receive either a 4-week The-Optimal-Lymph-Flow for Heart Failure (TOLF-HF) program or usual care alone. The primary outcome was the prevalence and burden of the fluid overload symptoms. Findings of the trial showed that the TOLF-HF intervention was effective in reducing the prevalence or burden of the majority of fluid overload symptoms. TOLF-HF intervention also demonstrated significant improvement in the outcomes of abnormal weight gains (MD: −0.82; 95% CI: −1.43 to −0.21; *P* = 0.010) and physical functions (*F* = 13.792, *P* < 0.001).

**Conclusions:**

The TOLF-HF program focusing on activating lymphatic system through the performance of therapeutic lymphatic exercises holds the promise as an adjuvant therapy for patients with heart failure to manage fluid overload symptoms, reduce abnormal weight gains, and improve physical functions. Future larger-scale study with longer duration of follow-up is needed.

**Clinical Trial Registration:**

http://www.chictr.org.cn/index.aspx, identifier ChiCTR2000039121.

## 1. Introduction

The increase in heart failure (HF) prevalence and considerable burden to healthcare systems have made HF a global pandemic (1, 2). Fluid overload, the hallmark of HF, refers to a progressive body fluid retention or redistribution that impedes multiple body system functions (3). Many symptoms are associated with fluid overload symptoms, such as dyspnea, coughing, wheezing, edema, pain, or fatigue (3, 4). These symptoms are highly prevalent and burdensome, leading to poor quality of life and decreased daily living functions (4). The experience of fluid overload symptoms not only is the most commonly cited reason for HF hospital admission or readmission but also creates substantial burdens on health system, patients, and families (5, 6).

Lymphatic system plays an essential role in body fluid regulation and pathogenesis of cardiovascular diseases (7–9). The lymphatic vascular system that lies parallel to the blood vascular system maintains fluid homeostasis between intravascular and interstitial spaces by collecting fluid from the interstitial space and draining it back into the venous circulation (10–12). It is estimated that up to 8 liters of interstitial fluid are scavenged each day by the lymphatic capillaries that line all organs and are transported by the unidirectional lymphatics to the blood circulation (10). Fluid overload occurs when fluid is not drained at the same rate as it leaks into the interstitial spaces (10, 11).

Activating lymphatic system holds a great promise for reducing fluid overload as the interstitial fluid is drained exclusively by lymphatic pumping (11, 12). Exercise that stimulates muscle contraction and breathing can promote lymphatic flow, and potentially decreases fluid accumulation in tissues and interstitium (12, 13). A three- to six-fold increase in the lymph clearance rates was observed using a scintigraphic device during human active exercise compared with resting levels (14). This phenomenon was also confirmed in an animal experiment using direct cannulation of a lymphatic duct in anaesthetized sheep (15). Therefore, therapeutic exercises focusing on optimizing lymph fluid flow can be promising to prevent and manage fluid overload symptoms in HF.

This pilot study was conducted to examine the effects of exercises stimulating lymphatic system on fluid overload symptoms in HF. We hypothesized that lymphatic exercises that simulate lymphatic pumping and drainage could decrease fluid accumulation in tissues and interstitium in patients with HF, in turn, alleviate fluid overload symptoms and burden (12, 13). We have developed and extensively tested a non-pharmacological *The-Optimal-Lymph-Flow* (TOLF) intervention that builds patients self-management skills to promote lymph fluid flow and results in reduced pain, swelling, lymph fluid level, reverse of mild lymphedema, and improved function and quality of life in cancer patients (16–24). TOLF includes strategies to promote lymph fluid flow: therapeutic lymphatic exercises, healthy diet, and proper sleep (16–24). The essential component of TOLF is 8-minute lymphatic exercises (i.e., muscle-tightening deep breathing, muscle-tightening pumping, and large muscle exercises) designed to stimulate lymphatic system by simulating lymphatic pumping to promote lymph fluid flow (16). We conducted a pilot single-blind two-group randomized controlled trial (RCT) to evaluate the preliminary effects of 4-week TOLF lymphatic exercise training in 66 adult patients with HF.

## 2. Materials and methods

### 2.1. Design and setting

*The-Optimal-Lymph-Flow for Heart Failure* (*TOLF-HF*) trial utilized a prospective, single-center, two-arm RCT design. This trial was conducted in the Department of Cardiology of West China Hospital, the largest national center for the diagnosis and treatment of complicated and critical cardiovascular diseases in western China. The Ethics Committee of West China Hospital, Sichuan University approved this trial (No. 2019-202) and this trial was registered on Chinese Clinical Trial Registry (ChiCTR2000039121). The current study is reported following the CONSORT reporting guideline (25).

### 2.2. Recruitment and participants

Potential participants were identified from March 2019 to January 2020 *via* review of inpatient census lists and were introduced to the study two days before their discharge. A total of 66 patients met all eligibility criteria and consented to study participation after the recruiting researcher explained the study objectives, procedures, and possible risks/benefits. If patients agreed to participate in the trial, they were asked to sign a written informed consent and complete baseline assessment.

Inclusion criteria for the trial included that patients were: (1) aged 18 to 80 years; (2) hospitalized with a primary diagnosis of HF; (3) classified as New York Heart Association (NYHA) functional class II or III; (4) willing to complete the home-based *TOLF-HF* program. The diagnosis of HF was made by an expert team of cardiologists in compliance with 2018 Chinese guidelines for the diagnosis and treatment of HF (26). We excluded patients who (1) had severe liver impairment (i.e., Child-Pugh score ≥10) or kidney insufficiency (i.e., an estimated glomerular filtration rate <30 ml/min/1.73 m^2^) or malignant tumors; (2) had a terminal condition with a life expectancy of less than 6 months; (3) had received or were waiting for heart transplantation; (4) were undergoing respiratory muscle training or resistance training; (5) were participating in other research programs; or (6) were unable to read or understand Chinese.

### 2.3. Randomization and masking

Following baseline assessment, participants were randomly assigned to receive either a 4-week TOLF-HF program plus usual care or usual care alone with a 1:1 ratio. Randomization assignment was carried out by an independent research assistant using random numbers generated by the random number generator in the SPSS for Windows (Version 22.0; IBM Corp., Armonk, NY, USA) and individual allocations were concealed in sequentially numbered, opaque and sealed envelopes until interventions were assigned. Because of the nature of the exercise intervention, blinding participants and interventionist was not feasible. However, the outcome assessor and data analyst were blinded to the group allocation.

### 2.4. Interventions

Patients allocated to the control group only received usual care. In addition to guideline-directed medical therapy (26, 27), usual care included provision of a written discharge summary (including the diagnosis, disease course, inhospital treatment record and postdischarge medications, etc.) and oral instructions on appropriate lifestyle behaviors and medication management by the ward nurse upon discharge, and patients might be referred to post-discharge support as needed. All patients were routinely scheduled to visit the specialist clinic 4 weeks after discharge. No other structured educational or supportive postdischarge care was provided.

Patients allocated to the intervention group received the 4-week *TOLF-HF* intervention in addition to usual care. *TOLF-HF* is a patient-centered behavioral program featuring self-care risk reduction strategies to promote lymph flow with the aim to improve fluid overload symptoms in patients with HF (16). Easy-to-learn self-care strategies in activating lymphatic system consists of muscle-tightening deep breathing and muscle-tightening pumping exercises, as well as large muscle exercises. Specifically, muscle-tightening deep breathing activates lymphatic ducts and facilitates lymph fluid drain, muscle-tightening pumping helps lymph fluid flow and reduce fluid build-up, and large muscle exercises enhance lymph fluid flow and drain across the whole body (16). The self-care strategies along with their corresponding physiological rationales are presented in Table 1.

**Table 1:** *TOLF-HF* program strategies and rationales (16, 18).

Strategies and exercises*	Rationales	Frequency & situations
Muscle-tightening deep breathing exercises	• The whole-body lymph fluid has to be drained through the lymphatic ducts above the heart. Muscle-tightening-deep-breathing stimulates lymphatic ducts and helps lymph fluid drain.	• At least twice a day in the morning & at night before brushing teeth or as much as the patient wants throughout the day
	• Lymph fluid drains when muscles move. Muscle-tightening-deep-breathing creates the whole-body muscle movements that create muscle milking and pumping action and help to drain lymph fluid	• Air-Travel: before take-off and after landing.
		• Sedentary lifestyle: At least every 4 h
Muscle-tightening pumping exercises	• Muscle-tightening pumping exercises create arm muscle pumping. This helps lymph fluid flow and decreases the fluid build-up in the upper and lower extremities.	• At least twice a day in the morning & at night before brushing teeth or as much as the patient wants throughout the day
	• Muscle-tightening pumping exercises build the muscles in upper and lower limbs that helps lymph fluid flow and drain.	• Air-Travel: before take-off and after landing
		• Sedentary lifestyle: At least every 4 h
Large muscle exercises: walking, marching at home, dancing, swimming, yoga, Tai Chi, etc.	• Large muscle exercises create muscle milking and pumping to promote overall body lymph fluid flow and drain.	• At least 10-minutes daily.
		• Air-Travel: get up and walk around for flight over 4 h.
		• Sedentary lifestyle: Get up and walk at least every 4 h.

* For step-by-step instruction for each exercise, please contact the corresponding author.

*TOLF-HF* program was initiated as soon as possible after the baseline assessment and within 48 h before hospital discharge. A trained researcher (Q.M.) delivered the intervention in a 30-minute one-to-one meeting. First, a step-by-step video presentation showing how exercise should be done and how often it should be done was demonstrated to achieve unambiguous and standardized instruction. Next, patients were asked to practice on their own, and the researcher observed and corrected their wrong movements in a timely manner until they could perform all the movements themselves. The researcher made a video recording while patients were performing the lymphatic exercises. The video clip was provided to patients for them to review whenever necessary. Family members were involved throughout the procedure and asked to accompany and encourage patients' practices at home. After hospital discharge, subsequent WeChat contacts were made every week by the researcher to identify any associated barriers, offer pertinent advice, and motivate adherence to the *TOLF-HF* protocol.

### 2.5. Outcomes

We operationalized the primary outcome of the fluid overload symptoms as the prevalence and burden of symptoms associated with fluid retention (4, 28). Fluid overload symptoms were evaluated using the Chinese version of the Memorial Symptom Assessment Scale—Heart Failure (MSAS-HF) (29–31) to include symptoms of shortness of breath, difficulty breathing when lying flat, cough, lack of energy, swelling of legs or ankles, and waking up breathless at night (31, 32). Participants first reported whether they experienced each listed symptom for the past week; if so, then they reported the frequency on a scale ranging from 1 (rarely) to 4 (almost constantly) and severity on a scale from 1 (mild) to 4 (very severe) (29, 30, 33). Symptom burden scores are calculated as the mean of the frequency and severity of each symptom, with higher scores indicating greater symptom burden (33). The reliability of the MSAS-HF has been previously confirmed in the Chinese context with a Cronbach's *α* coefficient of 0.946 (31).

The secondary outcomes included the number of abnormal weight gains and physical functions. An electronic scale and a structured daily log were provided to each participant to take home for recording body weight, and lymphatic exercise performance for participants in the *TOLF-HF* intervention group. All participants received the instructions on the use of the scale and self-monitor of weight daily at home. A rapid weight gain of greater than 1 kg in one day or greater than 2 kg in three days is considered as one occurrence of abnormal weight gain. Physical functions were measured using the physical subscale of the Minnesota Living with Heart Failure Questionnaire (MLHFQ) (34), which is one of the most commonly used disease-specific instruments that reflects how HF affects everyday life and functions of patients (35). The physical subscale of MLHFQ consists of 8 items rated on a 6-piont Likert scale from 0 (no impact) to 5 (very high impact). Summary scores range from 0 to 40; higher scores indicate poorer functioning. The Chinese version of the MLHFQ has been validated and the physical subscale demonstrated adequate reliability (*α* = 0.950) (36).

Adverse events were defined as any discomfort or injury induced by the *TOLF-HF* program during the intervention period. Participants were asked to report to the research assistant immediately if an adverse event occurred due to the exercises.

### 2.6. Data collection

All participants completed the outcome measures at baseline and 4 weeks post-intervention under the guidance of an unbiased research assistant who was unaware of the study hypothesis and the group allocation, and were required to share their weight diary with the assistant *via* WeChat every week. Sociodemographic and clinical information were collected *via* patient self-report and review of electronic medical records.

### 2.7. Sample size

PASS software 15.0 (NCSS, LLC, Kaysville, UT, USA) was used to estimate the sample size based on the primary outcome measure informed by the results of our preliminary trial, which showed a 35% difference (40% vs. 75%) in the prevalence of shortness of breath (the most prevalent fluid overload symptom) between the groups. To detect this expected between-group difference at a two-sided significance level of 0.05 with 80% power, we required data from at least 28 participants per group. Accounting for an anticipated attrition rate of 15%, the final sample size needed for the study was 33 patients per group, a total of 66 patients to be randomized into control and intervention groups.

### 2.8. Statistical analysis

The baseline characteristics of the study participants were calculated using descriptive analyses stratified by treatment group. Data normality was verified using the Shapiro-Wilk test. Means with standard deviations (SDs) or medians with inter-quartile ranges (IQRs) were estimated for the continuous variables based on the normality of the data distribution. Differences between intervention and control groups were estimated using the independent sample t-test for normally distributed variables and the Mann-Whitney U test for variables that did not conform to normal distribution. Where appropriate, analysis of covariance (ANCOVA) was also calculated to adjust for baseline outcome measures and important demographics. Counts with percentages were calculated for the categorical variables. Pearson's *χ*^2^ test with continuity correction or Fisher's exact probability test were used for between group differences. The modified Poisson regression models were built to estimate the adjusted risk ratios (RRs) with 95% confidence intervals (CIs) to estimate the intervention effect of *TOLF-HF* program on symptom prevalence while controlling for baseline covariates. All statistical analyses were conducted using SPSS 26.0 for Windows (IBM Corp., Armonk, NY, USA). A two-sided *P* value of less than 0.05 was set for significance level.

## 3. Results

### 3.1. Study participants

**Figure 1:**
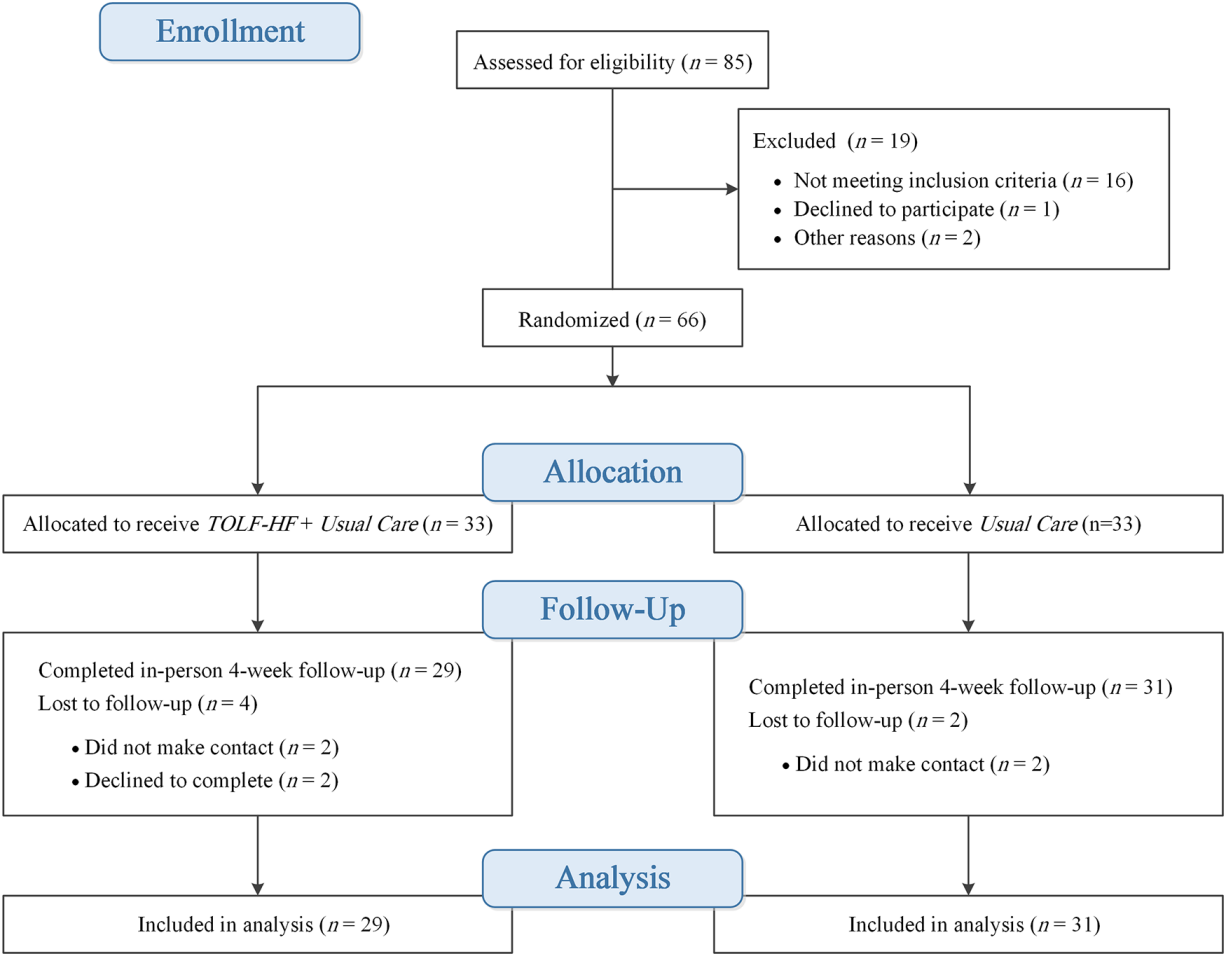
Patient flow diagram.

The CONSORT flow diagram is shown in Figure 1. Eight-five patients were screened for eligibility; 66 patients met the inclusion criteria. Only six patients (9%) were not be able to complete the study, despite the final stage of the study being challenged by the COVID-19 outbreak. Thus, 60 patients completed both the baseline and follow-up assessments; data from the 60 patients were used for the analysis.

Of the 60 patients, the mean age was 59.92 in years [standard deviation (SD) = 12.13] and 35% (*n* = 21) were female. The mean left ventricular ejection fraction (LVEF) for the participants was 34.63 (SD = 16.19); 48.3% of the participants were classified as NYHA class II and 51.7% class III. Detailed participant characteristics are presented in Table 2. Baseline characteristics between groups were similar, except for the number of co-morbidities: patients in *TOLF-HF* group suffered more co-morbidities as compared with control group (*Z* = 2.449, *P* = 0.014).

**Table 2:** Baseline characteristics of study participants.

Variables	*TOLF-HF* group (*n *= 29)	Control group (*n *= 31)	*t*/*χ*^2^/*Z*	*df*	*P* value
Age, mean (SD), years	58.07 (12.79)	61.65 (11.42)	−1.144	58	0.257
Gender, *n* (%)*			0.000	1	1.000
Male	19 (65.5)	20 (64.5)			
Female	10 (34.5)	11 (35.5)			
Level of education, *n* (%)^†^			1.854	3	0.603
No formal education	4 (13.8)	2 (6.5)			
Elementary school	6 (20.7)	8 (25.8)			
Secondary school	10 (34.5)	8 (25.8)			
College/university and above	9 (31.0)	13 (41.9)			
Occupational status, *n* (%)*			0.071	1	0.789
Employed	16 (55.2)	15 (48.4)			
Unemployed	13 (44.8)	16 (51.6)			
Caregiver, *n* (%)^†^			4.447	3	0.206
Self (no caregiver)	0 (0)	2 (6.5)			
Spouse/partner	5 (17.2)	1 (3.2)			
Offspring	12 (41.4)	15 (48.4)			
Paid caregiver	12 (41.4)	13 (41.9)			
Weight, mean (SD), kg	60.27 (12.24)	58.97 (15.80)	0.347	56	0.730
Height, mean (SD), cm	165.11 (8.35)	164.78 (8.42)	0.153	56	0.879
BMI, mean (SD), km/m^2^	22.04 (3.76)	21.72 (5.27)	0.266	56	0.791
SBP, mean (SD), mmHg	110.76 (20.85)	114.07 (17.57)	−0.666	58	0.508
DBP, mean (SD), mmHg	75.90 (16.07)	76.19 (12.93)	−0.079	58	0.937
HR, mean (SD), bpm	91.00 (21.49)	89.35 (17.40)	0.327	58	0.745
Length of hospital stay, mean (SD), days	10.28 (4.20)	9.52 (2.55)	0.853	58	0.397
Duration of HF, median (IQR), years^‡^	2.00 (0.00–6.00)	1.00 (0.00–4.00)	0.169		0.865
HF type, *n* (%)*			0.000	1	1.000
Left heart failure	15 (51.7)	16 (51.6)			
Right heart failure	14 (48.3)	15 (48.4)			
Underlying diseases, *n* (%)^†^			1.473	4	0.914
Cardiomyopathy	15 (51.7)	20 (64.5)			
Valvular heart disease	6 (20.7)	5 (16.1)			
Hypertension	4 (13.8)	3 (9.7)			
Coronary heart disease	3 (10.3)	2 (6.5)			
Others	1 (3.4)	1 (3.2)			
Number of co-morbidities, median (IQR)^‡^	2.0 (1.0–3.0)	1.0 (0.0–2.0)	2.449		**0** **.** **014**
LVEF, median (IQR), %^‡^	27.0 (22.5–38.5)	35.0 (23.0–44.0)	–1.066		0.286
NYHA function class, *n* (%)*			1.693	1	0.193
Grade II	18 (62.1)	13 (41.9)			
Grade III	11 (37.9)	18 (58.1)			
NT-proBNP, median (IQR), pg/ml^‡^	1,626.0 (673.0–8,127.0)	3,075.0 (1,389.5–4,863.5)	−1.123		0.261
Dose of diuretics, median (IQR), mg/day^‡^	80.0 (40.0–120.0)	40.0 (40.0–80.0)	1.341		0.180

*df*, degree of freedom; SD, standard deviation; BMI, body mass index; SBP, systolic blood pressure; DBP, diastolic blood pressure; HR, heart rate; HF, heart failure; IQR, inter-quartile range; LVEF, left ventricular ejection fraction; NYHA, New York Heart Association (functional class); NT-proBNP, N-terminal pro-brain natriuretic peptide.

* The data were compared by Pearson's *χ*^2^ test with continuity correction.

^†^ The data were compared by Fisher's exact probability test.

^‡^ The data were expressed as median (IQR) and analyzed by the Mann-Whitney U test.

### 3.2. Primary outcome: fluid overload symptoms

#### 3.2.1. The prevalence of fluid overload symptoms

**Figure 2:**
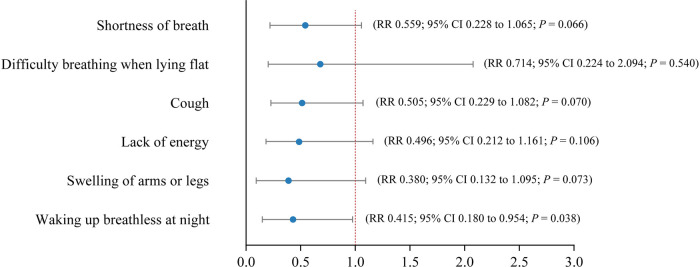
Forest plot showing estimates of RRs with 95% CIs of symptom prevalence between the *TOLF-HF* intervention and control groups after controlling for baseline prevalence values and the number of co-morbidities. RR, risk ratio; CI, confidence interval.

As shown in Table 3, no significant between-group differences at baseline were detected for symptom prevalence across the symptoms. At the study endpoint of week 4, the prevalence of shortness of breath (31.0% vs. 61.3%; *P* = 0.037) and swelling of arms or legs (13.8% vs. 41.9%; *P* = 0.033) were significantly lower in the *TOLF-HF* group than in the control group. The modified Poisson regression results shown in Figure 2 indicated that *TOLF-HF* intervention led to a significant reduction in the prevalence of waking up breathless at night (RR: 0.415; 95% CI: 0.180–0.954; *P* = 0.038) and showed trends towards decreasing the prevalence of shortness of breath (RR: 0.559; 95% CI: 0.288–1.065; *P* = 0.066), cough (RR: 0.505; 95% CI: 0.229–1.082; *P* = 0.070), and swelling of arms or legs (RR: 0.380; 95% CI: 0.132–1.095; *P* = 0.073), but did not reach statistical significance. No significant effects were found for difficulty breathing when lying flat (*P* = 0.540) or lack of energy (*P* = 0.106).

**Table 3:** Comparison of symptom prevalence at baseline and week 4 between the groups.

Symptom	Total prevalence	*TOLF-HF* group (*n *= 29), *n* (%)	Control group (*n *= 31), *n* (%)	*χ* ^2^	*P* value*
**Shortness of breath**					
Baseline	45 (75.0)	20 (69.0)	25 (80.6)	0.556	0.456
Week 4	28 (46.7)	9 (31.0)	19 (61.3)	4.362	0.037
**Difficulty breathing when lying flat**					
Baseline	44 (73.3)	20 (69.0)	24 (77.4)	0.201	0.654
Week 4	11 (18.3)	4 (13.8)	7 (22.6)	0.297	0.586
**Cough**					
Baseline	37 (61.7)	17 (58.6)	20 (64.5)	0.041	0.839
Week 4	22 (36.7)	7 (24.1)	15 (48.4)	2.822	0.093
**Lack of energy**					
Baseline	31 (51.7)	15 (51.7)	16 (51.6)	0.000	1.000
Week 4	19 (31.7)	6 (20.7)	13 (41.9)	2.221	0.136
**Swelling of arms or legs**					
Baseline	30 (50.0)	11 (37.9)	19 (61.3)	2.403	0.121
Week 4	17 (28.3)	4 (13.8)	13 (41.9)	4.540	0.033
**Waking up breathless at night**					
Baseline	26 (43.3)	11 (37.9)	15 (48.4)	0.309	0.578
Week 4	18 (30.0)	6 (20.7)	12 (38.7)	1.538	0.215

* *P* values were derived from the Pearson's *χ*^2^ test with continuity correction.

#### 3.2.2. The burden of fluid overload symptoms

As summarized in Table 4, no significant differences were observed at baseline in terms of median burden scores for all investigated symptoms between the two groups. After the intervention, the *TOLF-HF* group had significantly lower median burden scores than the control group for shortness of breath (Med*_TOLF−HF_*_ _= 0, IQR 0–1 vs. Med_control _= 1, IQR 0–2; *P* = 0.015), cough (Med*_TOLF−HF_*_ _= 0, IQR 0–1 vs. Med_control _= 1.25, IQR 0–2; *P* = 0.040), and swelling of arms or legs (Med*_TOLF−HF_*_ _= 0, IQR 0–1 vs. Med_control _= 1, IQR 0–1.5; *P* = 0.021). While no significant differences were found for difficulty breathing when lying flat (*P* = 0.297), lack of energy (*P* = 0.136), or waking up breathless at night (*P *= 0.227).

**Table 4:** Comparison of symptom burden at baseline and week 4 between the groups.

Symptoms	*TOLF-HF* group (*n *= 29), median (IQR)	Control group (*n *= 31), median (IQR)	*Z*	*P* value*
**Shortness of breath**				
Baseline	2.50 (1.50–3.00)	2.00 (1.50–3.00)	0.079	0.937
Week 4	0.00 (0.00–1.00)	1.00 (0.00–2.00)	−2.439	0.015
**Difficulty breathing when lying flat**				
Baseline	2.00 (1.00–3.00)	2.50 (1.00–3.00)	−0.821	0.412
Week 4	0.00 (0.00–0.00)	0.00 (0.00–1.00)	−1.042	0.297
**Cough**				
Baseline	2.00 (1.50–3.00)	2.00 (1.00–3.00)	−0.191	0.848
Week 4	0.00 (0.00–1.00)	1.25 (0.00–2.00)	−2.052	0.040
**Lack of energy**				
Baseline	2.00 (1.00–3.00)	2.00 (0.00–2.50)	−0.071	0.943
Week 4	0.00 (0.00–1.00)	1.00 (0.00–1.50)	−1.492	0.136
**Swelling of arms or legs**				
Baseline	2.75 (1.25–4.00)	3.00 (0.50–3.75)	−1.852	0.064
Week 4	0.00 (0.00–1.00)	1.00 (0.00–1.50)	−2.305	0.021
**Waking up breathless at night**				
Baseline	1.50 (0.00–2.38)	1.50 (0.00–2.50)	−0.983	0.326
Week 4	0.00 (0.00–1.38)	0.50 (0.00–1.00)	−1.209	0.227

IQR, inter-quartile range.

* *P* values were derived from the Mann–Whitney *U-*test.

### 3.3. Secondary outcomes

#### 3.3.1. The number of abnormal weight gains

**Figure 3:**
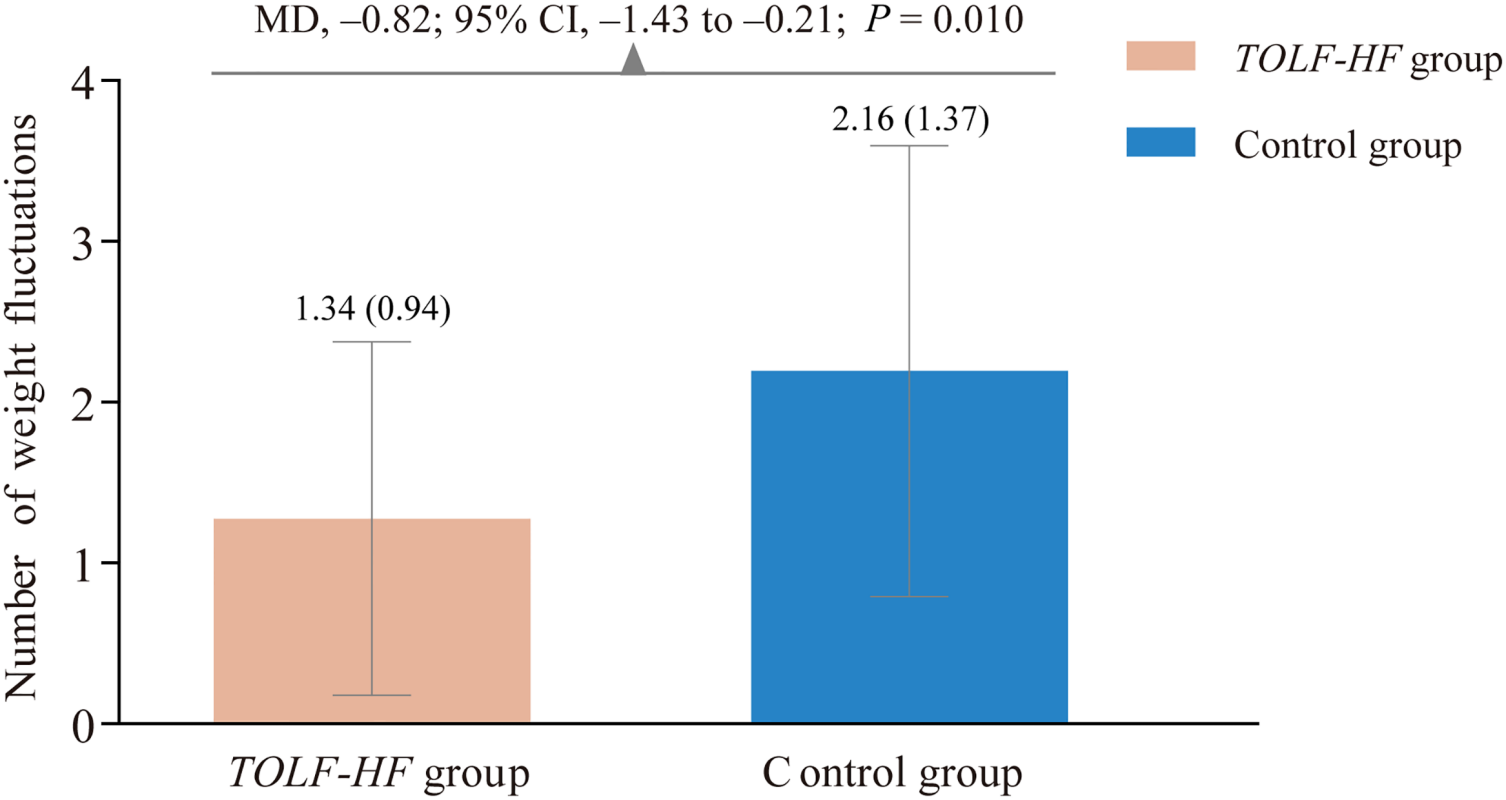
Column bar graph showing the comparison of the number of abnormal weight gains between the *TOLF-HF* intervention and control groups. MD, mean difference; CI, confidence interval.

A total of 39 times and 67 times of abnormal weight gains occurred during the study period in the intervention and control groups, respectively. As displayed in Figure 3, there was a significant between-group difference in favor of the *TOLF-HF* intervention group (MD: −0.82; 95% CI: −1.43 to −0.21; *P* = 0.010). Specifically, patients who received *TOLF-HF* intervention experienced 0.82 fewer times of rapid abnormal weight gain per person than those who only received care as usual.

#### 3.3.2. Physical functions

As presented in Table 5, no significant group differences were found in the baseline values for physical functions. At the endpoint of the study, the MLHFQ physical subscale scores were significantly lower in the intervention group compared to the control group (*F* = 1,792, *P* < 0.001), indicating better physical functions for patients received the *TOLF-HF* intervention.

**Table 5:** Comparison of physical functions at baseline and week 4 between the groups.

Physical functions	*TOLF-HF* group (*n *= 29), mean (SD)	Control group (*n *= 31), mean (SD)	*t/F*	*P* value
Baseline*	20.38 (10.42)	21.68 (9.44)	−0.506	0.614
Week 4^†^	9.93 (5.35)	16.55 (8.10)	13.792	<0.001

SD, standard deviation.

* The data were compared by the independent sample *t*-test.

^†^ The data were compared by analysis of covariance (ANCOVA) after controlling for the pre-intervention physical functioning values and the number of co-morbidities.

### 3.4. Adverse events

No exercise-related adverse events occurred throughout the course of the study. The *TOLF-HF* program was safe for patients who were discharged from the hospital with HF.

## 4. Discussion

The fundamental function of the lymphatic system in fluid homeostasis has long been overlooked, largely because of the difficulty in visualizing the transparent lymphatic vessels (8). This pilot clinical trial was the first to provide initial empirical evidence that the *TOLF-HF* program focusing on stimulating lymphatic system to promote fluid flow was effective in alleviating fluid overload symptoms of shortness of breath, cough, swelling of arms or legs and waking up breathless at night, decreasing abnormal weight gains, and improving physical functions for patients with HF. Recent research advances into the lymphatic network and the pathogenesis of cardiovascular diseases have made the lymphatic circulation an active target for the treatment of cardiovascular diseases (8, 9). The innovative application of *TOLF-HF* lymphatic exercises in patients with HF may open new avenues for nonpharmaceutical interventions for prevention and management of fluid overload in HF.

The accumulation of body fluid is an important driver of disease progression and symptom deterioration in HF (5). Worsening HF triggered by volume overload would manifest as multiple congestion symptoms including symptoms of pulmonary congestion and systemic venous congestion, causing patient distress (4, 28). We found that the 4-week *TOLF-HF* lymphatic exercises in addition to standard patient care was beneficial in reducing either the prevalence or burden of the majority of the fluid overload symptoms. Specifically, patients who received the intervention had 64.3% less likelihood of reporting waking up breathless at night and a trend towards less shortness of breath, cough, and swelling of arms or legs. Patients also benefited from the intervention by an alleviated symptom burden for shortness of breath, cough, and swelling of arms or legs. The symptomatic relief of trouble breathing (i.e., shortness of breath and waking up breathless at night) and coughing can be explained by the improved congestion in the pulmonary circulation while the relief of limb swelling can be explained by the improved congestion in the systemic circulation (37, 38). The core *TOLF-HF* intervention consists of lymphatic exercises of muscle tightening, stretching and pumping movements coordinated with deep breathing to imitate the physiological process of lymph pumping and propulsion; in addition, lymphatic exercises also induce musculoskeletal contractions, arterial pulsations, skin tensions, postural changes, and breathing alterations. Such lymphatic exercises could produce a synergistic effect to accelerate fluid volume removal not only in the thoracic area but also throughout the whole body, thus achieving positive outcomes in fluid overload symptom relief (16).

Our study found the *TOLF-HF* exercises did not reduce neither the prevalence nor the burden of difficulty breathing when lying flat or lack of energy. Difficulty breathing when lying flat represents the deteriorated cardiac health status and occurred more frequently in patients with more severe HF (39), while all patients recruited for this study were in chronic or stable phase and classified as NYHA class II or III. This might help explain the nonsignificant result for this symptom within our study. Lack of energy (also known as exercise intolerance or fatigue) in HF is a complex and multifactorial phenomenon; the exact pathogenesis of this symptom is not yet fully understood. On the one side, lack of energy is indicative of skeleton muscle hypoperfusion caused by interstitial fluid overload and poor cardiac output (38, 39). On the other side, lack of energy also reflects clinical factors such as aging, anemia, muscle weakness, nutritional deficiencies, sleep disorders, and psychosocial distress (40, 41). Therefore, it is reasonable that *TOLF-HF* exercises particularly targeting on the problem of fluid overload may not be as effective as expected in attenuating the perception of lack of energy.

Weight fluctuation is an objective and measurable indicator of fluid volume status in HF (42). Our study suggested that the *TOLF-HF* intervention was effective in reducing the number of abnormal weight gains. Sudden weight gain commonly means that excess fluid is building up in the body and signals the worsening of cardiac pumping capacity (42). This finding supported our hypothesis that the *TOLF-HF* intervention, designed to stimulate the lymphatic system to promote fluid flow, was effective to decrease or offset the water retention in the body. The *TOLF-HF* intervention is safe and easy to perform while patients are sitting, standing, or lying; this makes easier for patients to adhere to the lymphatic exercises. Thus, the *TOLF-HF* intervention holds a great promise as an adjuvant intervention for patients with HF to maintain body weight within a stable range.

In addition, physical functions are important outcomes that reflects the impact of HF on patients' functional status (35). Findings of the trial demonstrated that the *TOLF-HF* intervention had significantly improved physical functions for patients with HF. The positive effect can be explained by the beneficial role of *TOLF-HF* exercises in ameliorating fluid overload symptoms (30, 43) and the fact that the intervention prompted the patients to lead an active lifestyle. It is well-known that adopting a physically active lifestyle is one of the best ways that people can do to help prevent illness, preserve health, and enhance physical functions (44, 45).

To our knowledge, previous studies focusing on the effects of exercise intervention on fluid overload in HF were limited (46). This is a pioneer study that examined exercise-based intervention specifically targeting lymphatic system on alleviation of fluid overload symptoms by providing rigorous prospective randomized controlled data. Efficacious lymphatic transport entails the coordination of lymphatic pumping. Specifically, lymphatic contractions occur with a phase lag as the contractions propagated downstream from one lymphangion to the next generated the highest lymph flow (7, 10). The *TOLF-HF* exercises produced the desired effects as the intervention was designed to mimic the physiological process of lymphatic pumping (16). Apart from that, this intervention had a number of strengths. First, the *TOLF-HF* intervention is easy-to-learn and elderly-friendly, as such the intervention is especially appropriate for HF population. Second, the *TOLF-HF* intervention consists of a series of relatively low-intensity exercises and requires low physical demands. Even immobilized or bedridden patients can perform the most parts of the exercises. Finally, the *TOLF-HF* intervention has been shown to be feasible and acceptable to patients in this pilot trial as well as in previous experimental studies with breast cancer survivors (16–18, 24). Therefore, the *TOLF-HF* intervention is a pragmatic, effective, well-tolerated, and easily-integrated-into-daily-routine self-care program for the prevention and management of fluid overload and associated symptoms in patients with HF.

We recognize that there are several limitations for the current study. There were no significant between-group differences in baseline characteristics except for the number of co-morbidities, some discrepancies still existed. Because of the relatively small sample size and the exploratory nature of this study, the possibility of chance findings cannot be excluded. Additional limitations include relatively short follow-up period, inclusion of the patients who had NYHA class II or III. Multicenter studies with a larger sample size, longer follow-up, and comprising of a wider spectrum of HF cases are needed. Patient participation and compliance with the exercise protocols are critical to fully elucidate the efficacy of the intervention. In the study, nearly 90% (26/29) patients reported performing the *TOLF-HF* exercises twice a day as prescribed. However, lack of real-time monitoring of the truly implemented exercise dose, limiting the study's ability to explore the dose-effectiveness of the intervention. Future technology innovation should focus on wearable device to monitor the adherence to *TOLF* lymphatic exercises.

In conclusion, our study suggests that the *TOLF-HF* program focusing on promoting lymph fluid flow through the performance of therapeutic lymphatic exercises is an efficacious adjuvant therapy for patients with HF in the management of fluid overload symptoms, maintenance of stable body weight, and improvement of physical functions. Given its acceptability, practicality, safety, and effectiveness, the *TOLF-HF* program should be further tested as part of routine HF care. The current study represents an important initial step targeting the lymphatic system to counteract fluid accumulation and symptom exacerbations. Future research is to develop wearable devices for real-time monitor of the home-based *TOLF*-*HF* protocol, such innovation may allow detailed elucidation of the mechanisms of lymphatic system stimulation on fluid overload symptoms and body fluid level in HF.

## Data Availability

The raw data supporting the conclusions of this article will be made available by the authors, without undue reservation.

## References

[1] Savarese G Lund LH. Global public health burden of heart failure. Card Fail Rev. (2017) 3:7–11. 10.15420/cfr.2016:25:2 28785469 PMC5494150

[2] Ziaeian B Fonarow GC. Epidemiology and aetiology of heart failure. Nat Rev Cardiol. (2016) 13:368–78. 10.1038/nrcardio.2016.25 26935038 PMC4868779

[3] Miller WL. Fluid volume overload and congestion in heart failure: time to reconsider pathophysiology and how volume is assessed. Circ Hear Fail. (2016) 9:e002922. 10.1161/CIRCHEARTFAILURE.115.002922 27436837

[4] Lee YW Jeng YJ Huang LH. Development and testing of a scale to assess fluid overload symptoms. Appl Nurs Res. (2015) 28:206–9. 10.1016/j.apnr.2014.10.001 25457273

[5] Pellicori P Kaur K Clark AL. Fluid management in patients with chronic heart failure. Card Fail Rev. (2015) 1:90–5. 10.15420/cfr.2015.1.2.90 28785439 PMC5490880

[6] Cooper LB Lippmann SJ DiBello JR Gorsh B Curtis LH Sikirica V The burden of congestion in patients hospitalized with acute decompensated heart failure. Am J Cardiol. (2019) 124:545–53. 10.1016/j.amjcard.2019.05.030 31208702

[7] Scallan JP Zawieja SD Castorena-gonzalez JA Davis MJ. Lymphatic pumping: mechanics, mechanisms and malfunction. J Physiol. (2016) 20:5749–68. 10.1113/JP272088 PMC5063934 27219461

[8] Aspelund A Robciuc MR Karaman S Makinen T Alitalo K. Lymphatic system in cardiovascular medicine. Circ Res. (2016) 118:515–30. 10.1161/CIRCRESAHA.115.306544 26846644

[9] Klaourakis K Vieira J Riley PR. The evolving cardiac lymphatic vasculature in development, repair and regeneration. Nat Rev Cardiol. (2021) 18:368–79. 10.1038/s41569-020-00489-x 33462421 PMC7812989

[10] Moore JE Jr. Bertram CD. Lymphatic system flows. Annu Rev Fluid Mech. (2018) 50:459–82. 10.1146/annurev-fluid-122316-045259 29713107 PMC5922450

[11] Itkin M Rockson SG Burkhoff D. Pathophysiology of the lymphatic system in patients with heart failure: jACC state-of-the-art review. J Am Coll Cardiol. (2021) 78:278–90. 10.1016/j.jacc.2021.05.021 34266581

[12] Stücker O Pons-Himbert C Laemmel E. Towards a better understanding of lymph circulation. Phlebolymphology. (2008) 15:31–6.

[13] Lane K Worsley D McKenzie D. Exercise and the lymphatic system: implications for breast-cancer survivors. Sport Med. (2005) 35:461–71. 10.2165/00007256-200535060-00001 15974632

[14] Havas E Parviainen T Vuorela J Toivanen J Nikula T Vihko V. Lymph flow dynamics in exercising human skeletal muscle as detected by scintography. J Physiol. (1997) 504:233–9. 10.1111/j.1469-7793.1997.233bf.x 9350633 PMC1159951

[15] Coates G O’Brodovich H Goeree G. Hindlimb and lung lymph flows during prolonged exercise. J Appl Physiol. (1993) 75:633–8. 10.1152/jappl.1993.75.2.633 8226462

[16] Fu MR Axelrod D Guth AA Cartwright F Qiu Z Goldberg JD Proactive approach to lymphedema risk reduction: a prospective study. Ann Surg Oncol. (2014) 21:3481–9. 10.1245/s10434-014-3761-z 24809302 PMC4163073

[17] Fu MR McTernan ML Qiu JM Ko E Yazicioglu S Axelrod D The effects of kinect-enhanced lymphatic exercise intervention on lymphatic pain, swelling, and lymph fluid level. Integr Cancer Ther. (2021) 20:153473542110267. 10.1177/15347354211026757 PMC8226364 34160294

[18] Fu MR Axelrod D Guth AA Scagliola J Rampertaap K El-Shammaa N A web- and mobile-based intervention for women treated for breast cancer to manage chronic pain and symptoms related to lymphedema: results of a randomized clinical trial. JMIR Cancer. (2022) 8:e29485. 10.2196/29485 35037883 PMC8893593

[19] Liu F Li F Fu MR Zhao Q Wang Y Pang D Self-management strategies for risk reduction of subclinical and mild stage of breast cancer-related lymphedema: a longitudinal, quasi-experimental study. Cancer Nurs. (2021) 44:E493–502. 10.1097/NCC.0000000000000919 34694088

[20] Fu MR Axelrod D Guth AA Rampertaap K El-Shammaa N Hiotis K Mhealth self-care interventions: managing symptoms following breast cancer treatment. mHealth. (2016) 2:28. 10.21037/mhealth.2016.07.03 27493951 PMC4970761

[21] Fu MR Axelrod D Guth AA Wang Y Scagliola J Hiotis K Usability and feasibility of health IT interventions to enhance self-care for lymphedema symptom management in breast cancer survivors. Internet Interv. (2016) 5:56–64. 10.1016/j.invent.2016.08.001 28255542 PMC5328240

[22] Chiang AT Chen Q Wang Y Fu MR. Kinect-based in-home exercise system for lymphatic health and lymphedema intervention. IEEE J Transl Eng Health Med. (2018) 6:4100313. 10.1109/JTEHM.2018.2859992 30456001 PMC6237707

[23] Chiang AT Chen Q Wang Y Fu MR. Motion sequence alignment for a kinect-based in-home exercise system for lymphatic health and lymphedema intervention. Annu Int Conf IEEE Eng Med Biol Soc. (2018):2072–5. 10.1109/EMBC.2018.8512635 30440810 PMC6241306

[24] Du X Li Y Fu L Chen H Zhang X Shui Y Strategies in activating lymphatic system to promote lymph flow on lymphedema symptoms in breast cancer survivors: a randomized controlled trial. Front Oncol. (2022) 12:1015387. 10.3389/fonc.2022.1015387 36353530 PMC9638430

[25] Moher D Hopewell S Schulz KF Montori V Gøtzsche PC Devereaux PJ CONSORT 2010 Explanation and elaboration: updated guidelines for reporting parallel group randomised trials. Br Med J. (2010) 340:c869. 10.1136/bmj.c869 20332511 PMC2844943

[26] Heart Failure Group of Chinese Society of Cardiology of Chinese Medical Association; Chinese Heart Failure Association of Chinese Medical Doctor Association; Editorial Board of Chinese Journal of Cardiology. Chinese Guidelines for the diagnosis and treatment of heart failure 2018. Zhonghua Xin Xue Guan Bing Za Zhi. (2018) 46:760–89. 10.3760/cma.j.issn.0253-3758.2018.10.004 30369168

[27] Yancy CW Jessup M Bozkurt B Butler J Casey DE Colvin MM 2017 ACC/AHA/HFSA focused update of the 2013 ACCF/AHA guideline for the management of heart failure: a report of the American college of cardiology/American heart association task force on clinical practice guidelines and the heart failure society of America. Circulation. (2017) 136:e137–161. 10.1161/CIR.0000000000000509 28455343

[28] Ambrosy AP Pang PS Khan S Konstam MA Fonarow GC Traver B, EVEREST Trial Investigators. Clinical course and predictive value of congestion during hospitalization in patients admitted for worsening signs and symptoms of heart failure with reduced ejection fraction: findings from the EVEREST trial. Eur Heart J. (2013) 34:835–43. 10.1093/eurheartj/ehs444 23293303

[29] Zambroski C Lennie T Chung M Heo S Smoot T Ziegler C. Use of the memorial symptom assessment scale-heart failure in heart failure patients. Circulation. (2004) 110(Supplement III(17)):739.

[30] Zambroski C Moser D Bhat G Ziegler C. Impact of symptom prevalence and symptom burden on quality of life in patients with heart failure. Eur J Cardiovasc Nurs. (2005) 4:198–206. 10.1016/j.ejcnurse.2005.03.010 15916924

[31] Guo J Lyu R Zhang J Wu X Ji S Li Z. Reliability and validity of the Chinese version of memorial symptom assessment scale-heart failure. Zhonghua Hu Li Za Zhi. (2014) 49:1448–52. 10.3761/j.issn.0254-1769.2014.12.008

[32] McDonagh TA Metra M Adamo M Gardner RS Baumbach A Böhm M, ESC Scientific Document Group. 2021 ESC guidelines for the diagnosis and treatment of acute and chronic heart failure: developed by the task force for the diagnosis and treatment of acute and chronic heart failure of the European society of cardiology (ESC) with the special contribution of the heart failure association (HFA) of the ESC. Eur Heart J. (2021) 42:3599–726. 10.1093/eurheartj/ehab368 34649282

[33] Son YJ Lee Y Song EK. Adherence to a sodium-restricted diet is associated with lower symptom burden and longer cardiac event-free survival in patients with heart failure. J Clin Nurs. (2011) 20:3029–38. 10.1111/j.1365-2702.2011.03755.x 21707808

[34] Rector TS Kubo SH Cohn J. Validity of the Minnesota living with heart failure questionnaire as a measure of therapeutic response to enalapril or placebo. Am J Cardiol. (1993) 71:1106–7. 10.1016/0002-9149(93)90582-W 8475878

[35] Garin O Herdman M Vilagut G Ferrer M Ribera A Rajmil L Assessing health-related quality of life in patients with heart failure: a systematic, standardized comparison of available measures. Heart Fail Rev. (2014) 19:359–67. 10.1007/s10741-013-9394-7 23681849

[36] Ho CC Clochesy JM Madigan E Liu CC. Psychometric evaluation of the Chinese version of the Minnesota living with heart failure questionnaire. Nurs Res. (2007) 56:441–8. 10.1097/01.NNR.0000299849.21935.c4 18004191

[37] Watson RD Gibbs CR Lip GY. ABC Of heart failure. Clinical features and complications. Br Med J. (2000) 320:236–9. 10.1136/bmj.320.7229.236 10642237 PMC1117436

[38] American Heart Association. Heart failure signs and symptoms. Available at: https://www.heart.org/en/health-topics/heart-failure/warning-signs-of-heart-failure#.W09BCtgzZmA (Accessed August 11, 2022).

[39] Massie BM. Chapter 58—Heart failure: pathophysiology and diagnosis; Goldman's Cecil Medicine, In: Saunders WB, editor. Goldman's cecil medicine, 24th ed. Philadelphia, PA, USA: W.B. Saunders (2012). p. 295–303. ISBN 978-1-4377-1604-7.

[40] Fink AM Gonzalez RC Lisowski T Pini M Fantuzzi G Levy WC Fatigue, inflammation, and projected mortality in heart failure. J Card Fail. (2012) 18:711–6. 10.1016/j.cardfail.2012.07.003 22939040 PMC3485074

[41] Smith OR Michielsen HJ Pelle AJ Schiffer AA Winter JB Denollet J. Symptoms of fatigue in chronic heart failure patients: clinical and psychological predictors. Eur J Heart Fail. (2007) 9:922–7. 10.1016/j.ejheart.2007.05.016 17631047

[42] American Heart Association. Managing heart failure symptoms. Available at: https://www.heart.org/en/health-topics/heart-failure/warning-signs-of-heart-failure/managing-heart-failure-symptoms (Accessed May 22, 2022).

[43] Blinderman CD Homel P Billings JA Portenoy RK Tennstedt SL. Symptom distress and quality of life in patients with advanced congestive heart failure. J Pain Symptom Manage. (2008) 35:594–603. 10.1016/j.jpainsymman.2007.06.007 18215495 PMC2662445

[44] Cunningham C O’Sullivan R Caserotti P Tully MA. Consequences of physical inactivity in older adults: a systematic review of reviews and meta-analyses. Scand J Med Sci Sports. (2020) 30:816–27. 10.1111/sms.13616 32020713

[45] Li Y Su S Luo B Wang J Liao S. Physical activity and depressive symptoms among community-dwelling older adults in the COVID-19 pandemic era: a three-wave cross-lagged study. Int J Disaster Risk Reduct. (2022) 70:102793. 10.1016/j.ijdrr.2022.102793 35036301 PMC8744411

[46] Fu MR Li Y Conway C Masone A Fang J Lee C. The effects of exercise-based interventions on fluid overload symptoms in patients with heart failure: a systematic review and meta-analysis. Biomedicines. (2022) 10:1111. 10.3390/biomedicines10051111 35625848 PMC9138396

